# Quantifying the Predictive Power of Social Determinants of Health in Cardiovascular Disease and Type 2 Diabetes Progression Using XGBoost: Retrospective Cohort Study

**DOI:** 10.2196/80377

**Published:** 2026-07-09

**Authors:** Hielke Muizelaar, Marcel Haas, Maarten van Aken, Rimke Vos, Marco Spruit

**Affiliations:** 1 Department of Public Health and Primary Care, Leiden University Medical Center, Albinusdreef 2, Leiden, 2333ZA, The Netherlands, 31 715268444, 31 715142588; 2 Department of Internal Medicine, Haga Hospital, The Hague, The Netherlands; 3 Leiden Institute of Advanced Computer Science, Faculty of Science, Leiden University, Leiden, The Netherlands

**Keywords:** cardiometabolic disease, machine learning, social determinants of health, XGBoost, risk prediction, type 2 diabetes, cardiovascular disease, biomedical risk factors, quantification

## Abstract

**Background:**

Cardiovascular diseases (CVDs) and type 2 diabetes (DM2) are influenced not only by biomedical risk factors but also by social determinants of health (SDOH). While the inclusion of SDOH in predictive models is increasingly advocated, few studies have quantified their specific contribution in a high-risk clinical cohort using robust statistical and machine-learning approaches.

**Objective:**

This study aims to quantify the added predictive value of SDOH in predicting CVD or DM2 disease onset within 5 years, within 10 years, and at any time during follow-up among individuals already at elevated risk and to compare this added value across multiple modeling setups and frameworks.

**Methods:**

We used a large, linked dataset of over 58,000 inclusion events from the Extramural Leiden University Medical Center Academic Network data warehouse in the Netherlands, combining structured coded diagnosis and medication records from general practitioners with individual-level socioeconomic data from Statistics Netherlands. Individuals aged 30 years and older without prior DM2 or CVD were followed to assess disease progression. We trained Cox proportional hazards (CPH) and Extreme Gradient Boosting (XGBoost) models to predict progression to DM2 or CVD within 5 and 10 years and overall. All analyses were performed using the R programming language. Experiments included comparisons of Systematic Coronary Risk Evaluation 2, CPH, and XGBoost models; evaluation of time-bound and survival-based formulations; and quantification of SDOH impact using feature subset XGBoost models and Shapley additive explanations (SHAP)–based importance.

**Results:**

For the 5-year prediction of CVD or DM2, the combined XGBoost model using biomedical and SDOH predictors achieved an area under the receiver operating characteristic curve (AUC) of 0.738, significantly outperforming the biomedical-only model (AUC=0.728; *P*=.01) and the SDOH-only model (AUC=0.691; *P*<.001). For 10-year CVD prediction, XGBoost achieved an AUC of 0.729, outperforming CPH (AUC=0.718; *P*=.02) and Systematic Coronary Risk Evaluation 2 (AUC=0.697; *P*<.001). For overall event prediction, XGBoost again performed best (AUC=0.719), significantly higher than CPH (AUC=0.704; *P*<.001). SHAP analyses showed that biomedical predictors contributed most strongly on a per-feature basis, while a subset of SDOH variables, particularly income- and benefit-related indicators, provided complementary predictive signal and ranked among the most influential predictors.

**Conclusions:**

Incorporating SDOH improved the prediction of CVD and DM2 onset in a clinically defined high-risk cohort. Across hundreds of linked predictors, SDOH provided measurable incremental discrimination beyond biomedical risk factors, and income- and benefit-related variables ranked among the most influential features. SHAP analyses indicated that this added value was largely driven by a limited subset of highly informative social predictors. These findings support integrating structured SDOH into clinically actionable risk stratification models.

## Introduction

The risk of developing cardiovascular disease (CVD) and type 2 diabetes (DM2) is related to a combination of interrelated metabolic anomalies (eg, insulin resistance, dyslipidemia, and hypertension), which are themselves socially patterned [1-3]. This is closely aligned with the clinical metabolic syndrome (MetS), a group of interrelated risk factors such as obesity, hypertension, dyslipidemia, and insulin resistance that together raise the risk of chronic cardiometabolic diseases [3,4]. MetS is widely recognized as a predictor of both DM2 and CVD, with formal definitions established by institutions such as the World Health Organization, the Joint Interim Statement, and the National Cholesterol Education Program Adult Treatment Panel III [5,6]. Although MetS definitions are typically based on biometric thresholds such as waist circumference, such measurements are not consistently available in routine health care data. In real-world primary care settings, the identification of cardiometabolic risk often depends on structured diagnosis codes and prescription records.

Therefore, extensive work has been done on the development of prediction models for the onset of DM2 and CVD, focusing predominantly on biomedical risk factors such as BMI, blood pressure, lipid levels, and comorbidities [7]. For CVD, notable examples include Systematic Coronary Risk Evaluation (SCORE) [8] and QRESEARCH cardiovascular risk algorithm (QRISK)-like statistical models [9-11]. For DM2, these models include SCORE2-Diabetes [12,13], the Australian DM2 risk model [14], and the QDScore algorithm [15]. These models typically rely on traditional statistical techniques such as the widely used Cox proportional hazards (CPH) models [16] and generally do not explicitly include social determinants of health (SDOH), despite growing evidence that sociodemographic factors significantly influence the risk development and progression of CVD and DM2.

The exclusion of variables such as income, education level, ethnicity, and household structure has raised concerns about the applicability of the models across diverse populations. For example, a study evaluating the SCORE2 model’s performance in an ethnically and socioeconomically diverse Dutch cohort found that the model underestimated CVD risk, particularly among individuals of low socioeconomic position and the Surinamese minority group [17]. The researchers suggested that incorporating socioeconomic position and ethnicity into CVD risk models could improve prediction accuracy and lead to fairer health care interventions.

While widely used, traditional statistical approaches such as CPH may not fully capture the complexity of disease progression, particularly when incorporating large multidimensional datasets that include SDOH and medical determinants. In this context, machine learning (ML) can offer significant added value. Unlike conventional statistical models, ML algorithms are capable of handling nonlinear relationships and large sets of data with high dimensionality. In direct comparison, ML models have been shown to be able to outperform logistic regression and proportional hazards models for the prediction of cardiovascular risk, in the right circumstances [18-26]. Over the years, several alterations to the Cox model have been proposed to make it more robust toward a relatively large number of predictors, including regularization techniques such as Least Absolute Shrinkage and Selection Operator (LASSO), Ridge, and Elastic Net regression. These methods help by shrinking the regression coefficients, reducing overfitting, and enabling variable selection [27].

However, these alterations do not eliminate the need for a linear relationship between predictors and log-hazard. Furthermore, the models are still unable to detect interactions between variables, as these will be ignored if they are not explicitly prespecified. To overcome these limitations, ML approaches offer a more flexible alternative. These algorithms can process large, high-dimensional datasets and identify hidden patterns without requiring predefined relationships between variables. It should be said that these models are regarded to perform up to standard only when sufficient meaningful predictors and events are present to train the models, and traditional models have been shown to work comparably or better in low-dimensional contexts, which is an important argument in favor of traditional models, due to their low cost of implementation, ease of use, and better explainability [19,28-30].

To address the aforementioned challenges, we aimed to assess the predictive value of SDOH when estimating risks of disease progression to DM2 and CVD. This involved creating a dataset of primary and secondary care determinants and then comparing models that use only biomedical risk factors with those that use both medical and social factors, with a specific focus on understanding how the addition of SDOH influences performance. We evaluated traditional statistical approaches, such as the CPH model, alongside more advanced ML algorithms that can better accommodate complex, high-dimensional data. For quantifying the added value of SDOH, we focused on binary risk prediction within clinically actionable time windows, particularly 5-year risk, which balances decision relevance, data quality, and robustness to censoring. Survival models were included as benchmarking analyses rather than as primary analytical framework.

Through this work, we aim to clarify whether and to what extent SDOH provide incremental predictive value beyond established biomedical risk factors in routine care data. By benchmarking statistical and ML models on 5-year CVD prediction, we motivate the use of Extreme Gradient Boosting (XGBoost) for subsequent analyses and examine the added value of SDOH within clinically meaningful and implementable fixed-horizon risk prediction settings.

## Methods

### Ethical Considerations

This study was approved by the Scientific Committee of the Department of Public Health and Primary Care at the Leiden University Medical Center (LUMC; reference WSC-2023‐55/SP). Furthermore, the study *“*HealthBox, Metabolic Syndrome Risk Stratification Patient Segmentation*”* (reference 24‐3005) was reviewed by the non-WMO (Medical Research Involving Human Subjects Act) Review Committee of LUMC Division 3. The committee concluded that the study does not fall under the scope of the WMO and is therefore exempt from review by a Medical Ethics Review Committee. The non-WMO committee raised no objections to the study.

In addition, the study received approval from the Scientific Committee of the HagaZiekenhuis (reference T24-030), as well as the Scientific Committee of the Haaglanden Medisch Centrum (reference 2024‐039 HealthBox).

In accordance with the Dutch Medical Treatment Contracts Act, patients were informed about the use of their health data for scientific research. The institutions involved operate under an opt-out system, whereby patients can indicate that their data should not be used for research purposes. Only data from patients who did not opt out were included in this study. Because this was a retrospective secondary analysis of routinely collected health care and administrative data, and because the non-WMO Review Committee determined that the study was not subject to the WMO, additional individual informed consent was not required.

Health care data from the Extramural Leiden University Medical Center Academic Network (ELAN) data environment were linked to socioeconomic and administrative data from Statistics Netherlands (CBS; *Centraal Bureau voor de Statistiek*) and analyzed through the CBS Remote Access environment. This secure environment provides controlled access to pseudonymized data. Researchers had access only to pseudonymized and deidentified data, and no directly identifiable patient information was available to the researchers during analysis. No individual-level results are reported in this paper, and all outputs were checked to prevent disclosure of identifiable information. Data handling complied with institutional data governance procedures and applicable Dutch and European privacy regulations, including the General Data Protection Regulation.

No participants were recruited directly for this study, and no financial compensation or other incentives were provided.

### Data and Dataset

#### ELAN Infrastructure

The data for this study were retrieved from the ELAN data infrastructure [31,32], which comprises 734,000 individuals registered in practices across the province of South Holland, the Netherlands. The ELAN general practitioner (GP) records span from the year 2000 to 2023. However, for the purposes of our study, we include preinclusion data only up to 2018 to ensure that there exists a minimum of 5 years of follow-up time available to assess the occurrence of outcomes.

ELAN was developed as part of an initiative to facilitate health care research on the key challenges of sustainable health care in the Netherlands. ELAN includes routine health care data collected from GPs and multiple hospitals located in the province of South Holland, the Netherlands. These primary and secondary care records include diagnoses, laboratory test results, prescribed medications, and treatment histories, providing a rich source of clinical information. Furthermore, ELAN is linked at the individual level to national datasets from CBS, expanding its scope well beyond medical data.

By integrating medical and socioeconomic data at the individual level, ELAN provides researchers with a unique opportunity to explore how medical and social factors interact to influence health outcomes. This is particularly useful for complex multifactorial conditions, such as CVD and DM2, where environmental factors and access to health care services have been shown to significantly influence disease progression along with biological risk factors.

#### Participants

Patients were included in the dataset if they were aged 30 years or older at the time of entering the cohort and had been assigned one or more of the following International Classification of Primary Care (ICPC) diagnosis codes, corresponding to relevant conditions, excluding CVD or DM2 outcomes:

1. A91.05: glucose intolerance
2. T82: obesity
3. T83: overweight
4. W84.02: diabetes gravidarum
5. W81: gestational hypertension, preeclampsia, and hemolysis, elevated liver enzymes, low platelet count
6. T99.06: polycystic ovary syndrome
7. Y07: erectile dysfunction
8. A29.01: family history of diabetes
9. K85: elevated blood pressure
10. T93: elevated cholesterol levels

For each of these inclusion criteria, we extracted data registration dates per patient. Each inclusion event was recorded as a separate entry in the dataset, which means that a single patient can have multiple entries corresponding to different inclusion events.

We selected this event-based design rather than a single-record per-patient structure to avoid reliance on a single, potentially arbitrary entry moment for each patient. In routine care, CVD and DM2 risk factors are recorded at different time points, and the availability and values of predictors depend on when a patient enters the cohort. Restricting the dataset to a single record per patient would therefore require selecting one moment of available information, which may discard relevant measurements.

In addition, patients may qualify for the risk group through different inclusion events at different time points. If only one record per patient was used, this would assume a single entry point, potentially oversimplifying the patient’s risk profile and possibly leading to inaccurate risk estimation. By recording multiple inclusion events, we can independently assess the predictive value of different inclusion criteria, providing insight into which diagnostic codes or medications contribute most significantly to CVD or DM2 risk.

Because the event-based structure allows repeated observations per individual, inclusion events are not fully independent. To prevent biased estimation and optimistic performance due to within-patient correlation, all model development and evaluation procedures used train-test splits at the individual level, ensuring that no patient contributed inclusion events to both the training and test sets. To further test robustness to this design choice, we conducted sensitivity analyses using alternative dataset constructions in which only a single inclusion event per patient was retained (first or last inclusion event). These sensitivity analyses are described in the “Experiments section.

To ensure that our dataset only includes individuals at substantial risk of developing CVD or DM2, we excluded patients with a CVD or DM2 diagnosis that occurred prior to their first inclusion in the risk group. Furthermore, we considered these CVD and DM2 diagnoses as outcomes (events) that we aimed to predict per inclusion event. The following ICPC codes were used:

1. T90: diabetes mellitus (DM2)
2. K47: angina pectoris (CVD)
3. K75: myocardial infarction (CVD)
4. K76: other ischemic diseases (CVD)
5. K89: transient ischemic attack (CVD)
6. K90: cerebral infarction (CVD)
7. K92.01: peripheral artery disease (CVD)

Cardiovascular mortality was deliberately not included as a separate outcome in this study because the primary objective of our modeling framework is to support early, clinically actionable prevention rather than late-stage fatality prediction. From a preventive care perspective, the most meaningful target is the first occurrence of nonfatal CVD, as this represents the stage at which risk-modifying interventions can still meaningfully alter the disease trajectory. For the same reason, we also do not include heart failure as an outcome. To reduce the influence of competing mortality during follow-up, cohort inclusion was restricted to individuals aged ≤70 years at entry.

### Biomedical Determinants

Biomedical data were extracted from the electronic health records of the GPs and supplemented by secondary care data.

1. Height in cm and weight in kg, from which BMI can be derived. Obesity (BMI ≥30 kg/m²) is one of the strongest predictors of both DM2 and CVD, with extensive evidence linking excess weight to insulin resistance, hypertension, and dyslipidemia.
2. Hemoglobin A_1c_ in percentage: a marker of long-term blood glucose control. It has been shown to increase the risk of CVD at higher levels.
3. Blood glucose level in mmol/L: used to diagnose impaired fasting glucose and diabetes, as sustained hyperglycemia is a key indicator of insulin dysfunction.
4. Low-density lipoprotein (LDL) cholesterol in mmol/L: often referred to as “bad cholesterol,” high LDL levels (>3 mmol/L) are associated with increased CVD risk.
5. High-density lipoprotein (HDL) cholesterol in mmol/L: the “good cholesterol” that has a protective effect against CVD. Low HDL levels (<1 mmol/L in men and <1.2 mmol/L in women) are associated with a higher cardiovascular risk.
6. Triglycerides in mmol/L: elevated triglyceride levels (>1.7 mmol/L) are closely associated with metabolic syndrome, diabetes, and increased cardiovascular risk.
7. Total cholesterol level in mmol/L: a composite measure of LDL, HDL, and triglycerides, often used in CVD risk calculations.

For each inclusion event, we extracted the most recent laboratory values prior to that event, ensuring the dataset reflects the patient’s state at inclusion. Additionally, we captured the maximum historical value of each measurement before the study period to account for past extremes (eg, spikes in glucose or LDL) that may indicate long-term risk. We supplemented these GP-based laboratory values with hospital records from 2 ELAN-affiliated hospitals, located within ELAN’s operating area. Measurements from primary and secondary care were stored in separate columns, allowing models to adjust for potential biases between care-setting sources.

These laboratory values, combined with inclusion codes and dates, form the core of our biomedical dataset. However, since ELAN data are only available from the point of registration, individuals may have met the inclusion criteria before any records exist. Additionally, switching GPs can lead to batch data entries, creating nonexisting clustering of events.

### CBS Data

In this paper, results are based on calculations by LUMC in project number 9002 using nonpublic microdata from CBS. The linkage of microdata from CBS enabled access to national demographic information, mortality data, insurance records on prescription medications, and SDOH. These datasets include a wide range of indicators such as personal characteristics, household structure, socioeconomic position, and mortality records. Incorporating these variables allowed for a more comprehensive analysis of how medical and social factors jointly influence the progression to CVD or DM2. Specifically, we included variables such as the country of birth of the individual and their parents, migration generation, primary and spendable income, and household head, as well as household size and number of children.

### Outcomes SCORE2 and Cardiovascular Risk Models

The SCORE2 risk prediction algorithms were developed with the objective of estimating 10-year fatal and nonfatal risk of CVD in individuals without a prior diagnosis of CVD or DM2 [28]. Within SCORE2, key risk factors for CVD were recorded at the point of study inclusion, including age, sex, smoking status, blood pressure, and cholesterol. Although SCORE2 is primarily designed for cardiovascular risk assessment rather than for more comprehensive profiling, its models and outputs have been incorporated into broader cardiometabolic research, for example, by serving as input features in models applied to MetS patient populations [33-35].

SCORE2, like traditional CPH models, assumes a linear relationship between predictor variables and the log-hazard of CVD risk. This means that each risk factor is expected to have a fixed and proportional effect on the outcome, which can oversimplify complex nonlinear interactions between variables, if present. For example, the effect that income has on CVD risk may reach a maximum at a certain value, meaning risk does not increase beyond a certain (high) income threshold, indicating a strong nonlinear relationship that these models are not able to handle well. Similarly, the impact of education on CVD risk could vary depending on factors such as access to health care or dietary habits, which also introduce nonlinearity in risk prediction. ML approaches offer a promising alternative by being able to detect such nonlinear interactions and relationships.

### ML for Survival (XGBoost)

Among the various ML methods used in survival analysis research, tree-based ensemble models have been shown to be particularly effective, especially in settings with complex, high-dimensional predictor spaces [36]. One prominent class of such methods is gradient boosting, which concerns first fitting a weak classifier to the data at hand and then continuously fitting new classifiers with the goal of reducing the loss of the previous classifier as much as possible. This is done via the use of a technique called gradient descent, which is used iteratively to find the next step that minimizes the outcome of a certain function, in this case, the loss function that corresponds to the model error.

One of the most well-regarded implementations of gradient boosting is XGBoost [37], which has been widely adopted across various ML tasks, including disease progression modeling [38,39]. In XGBoost, every classifier along the way is a type of decision tree, for which features from the data are chosen based on a greedy approach where a split is made that reduces the error the most. This approach to feature selection, combined with regularization techniques to prevent overfitting and parallelization at the node level to enhance performance, makes XGBoost highly efficient and accurate.

XGBoost also ranks feature importance based on gain, coverage, and frequency metrics, allowing researchers to identify the most influential predictors. This can be used to quantify the difference in importance between biomedical risk factors and SDOH in determining the risk of disease progression.

For survival analysis, XGBoost can be extended by incorporating loss functions adapted to time-to-event data. These include the CPH loss function, which allows XGBoost to estimate hazard ratios similarly to Cox models, and the accelerated failure time loss function, which models survival time directly. Using these adaptations, XGBoost can handle censored data efficiently, particularly in high-dimensional, complex datasets where interactions between medical factors play a crucial role.

In addition to XGBoost, we evaluated random forest models as a complementary tree-based ML approach. Random forests construct ensembles of decision trees using bootstrap aggregation and random feature selection, providing a nonparametric baseline that captures nonlinear effects without relying on further boosting [40]. Including random forest allows us to assess whether observed performance differences compared to other models can be attributed to the boosting framework rather than to general tree-based modeling. Random forest results are therefore reported as a comparative baseline rather than a primary modeling approach.

### Experiments

#### Overview of Experiments

To systematically assess the predictive value of SDOH in disease progression to CVD or DM2, we conducted four linked prediction experiments:

1. 10-year CVD risk prediction: the first experiment served as a benchmark, focusing on the prediction of 10-year CVD risk. We compared 3 modeling approaches: the SCORE2 algorithm, a CPH model, and our random forest and XGBoost models.
2. Overall event prediction (CVD or DM2): in the second experiment, we applied CPH and XGBoost to a broader prediction task that involves predicting CVD or DM2 without a fixed time frame. This allowed us to evaluate the general risk of progression to CVD or DM2 and helped determine which model is most suitable for subsequent analyses, as we used the best-performing model in the remainder of our experiments.
3. Time-bound prediction tasks (5-year and 10-year): in the third experiment, to reflect practical clinical decision-making, we assessed predictive performance in fixed-time binary classification settings. Here, the outcome is defined as the occurrence of CVD or DM2 within 5 or 10 years after entry into the cohort. These experiments allowed us to evaluate how our prediction models perform across different time windows. From this experiment, we chose a clinically relevant model for use in the next experiment to evaluate the added value of SDOH.
4. Contribution of SDOH: finally, we explicitly investigated the impact of SDOH. We trained and evaluated a clinically relevant model from the third experiment on three distinct feature sets: (1) medical predictors only, (2) social predictors only, and (3) the top 10 medical and social predictors. This design enabled us to quantify the added value of social variables in predicting disease progression.

Together, these experiments provided a framework for understanding the predictive utility of SDOH in both survival-based and fixed-time risk prediction settings. In the next section, we will go over how we implemented and tested our models on our dataset.

#### Model Implementation and Evaluation

To carry out our experiments, we implemented 4 types of models: the SCORE2 risk algorithm, a CPH model, random forest, and the XGBoost ML model. This section elaborates on how each model was adapted and applied within our experimental framework.

All trainable models in this study were developed using a random split at the individual level, where 80% of the individuals in the dataset were used for training and the remaining 20% were reserved for model evaluation. Since our dataset is based on inclusion events, where each individual can have multiple events, splitting on the row level could result in data from the same person appearing in both the training and test sets. This would violate the assumption of independence between training and test data and could lead to overly optimistic performance estimates, as the model may effectively be “tested” on patterns it has already seen during training. This approach enhanced the generalizability and validity of our model evaluation and ensured that performance metrics more accurately reflect how well the model would perform on unseen individuals in practice. To ensure consistency between experiments, the same train-test split was applied across all models and prediction tasks.

In addition, we performed sensitivity analyses using a single-event-per-patient dataset (first and last inclusion event). These analyses were used to assess whether predictive performance was driven by repeated observations per individual rather than by model overfitting and are reported in the “Results” section. To distinguish dataset effects from model effects, we also performed cross-evaluation in which models trained on the multievent dataset were evaluated on single-event test sets and vice versa.

We applied the SCORE2 10-year CVD risk model in the first experiment using the medical variables available in our dataset. Where possible, the input variables were aligned with those required by SCORE2. Smoking status was not included because it was not consistently available in a structured form in ELAN. SCORE2 was therefore implemented using the subset of required predictors available in the dataset (age, sex, systolic blood pressure, total cholesterol, and HDL cholesterol). SCORE2 results should be interpreted as an approximate benchmark rather than a fully specified clinical implementation. For SCORE2, CVD events were defined according to the same criteria used throughout the study, ensuring consistency between experiments.

For the CPH model, we used a regularized Cox model with LASSO (L1) penalty, implemented via the *glmnet* package in R. The L1 penalty enabled feature selection, which was especially valuable given the high-dimensional nature of our dataset. The model was trained on our dataset with survival data, with time-to-event recorded in days and event status coded as 1 (event) or 0 (censored). Ten-fold cross-validation within the training set was used to select the optimal penalty parameter λ based on model deviance. Because *glmnet* does not support clustered variance estimation, we refit the final Cox model using the *coxph* function (*survival* package) and apply a patient-level clustering term to obtain more robust SEs that account for within-patient correlation arising from repeated inclusion events. For time-specific risk estimation (eg, 10-year CVD risk), we derived individual survival probabilities from the refit *coxph* model using the estimated baseline survival function and the individual linear predictor. Specifically, the baseline survival curve was obtained from the training data using *survfit*, and the predicted 10-year risk was computed as 1 – *S*(*t|x*) at *t*=10 years. These risk estimates were then used for the area under the receiver operating characteristic curve (AUC) evaluation. Separate models were trained for each prediction task, and model performance was evaluated on the common test set. For comparability across modeling frameworks, survival models were evaluated using AUC on the corresponding binary event end points by converting predicted survival probabilities to fixed-horizon event risks.

Random forest models were included as a comparative tree-based ensemble baseline. They were implemented using the *ranger* package in R and trained using the same feature sets and the same individual-level train-test split as the other models. Model performance was evaluated using the same discrimination metrics on the held-out test set.

XGBoost was implemented using the *xgboost* package in R. For time-to-event prediction tasks, we used the accelerated failure time survival objective with a log-logistic distribution. For binary classification tasks, such as overall event prediction and 5-year or 10-year disease onset, we used the “binary:logistic” objective. Hyperparameter tuning was performed using random search over the predefined grid shown in Table 1, combined with 10-fold cross-validation within the training set. For each sampled hyperparameter configuration, models were trained for up to 2000 boosting rounds while monitoring cross-validated performance at each iteration. Early stopping was applied such that training was terminated if AUC did not improve for 50 consecutive rounds. The optimal number of boosting rounds was selected based on the best cross-validated performance, after which a final model was trained on the full training set using the selected hyperparameters and number of rounds, again using early stopping rounds on a validation subset of the training set (20%). After training, the model is evaluated on the held-out test set.

**Table 1.** Hyperparameter grid used for XGBoost^a^ random search^b^.

Hyperparameter	Values	Survival	Binary
aft_loss_distribution	“loglogistic,” “normal,” “extreme”	✓	
aft_loss_distribution_scale	1.0, 1.5, 2.0	✓	
max_depth	3, 6, 9	✓	✓
eta (learning rate)	0.01, 0.1	✓	✓
subsample	0.8, 1.0	✓	✓
colsample_bytree	0.8, 1.0	✓	✓
min_child_weight	1, 5	✓	✓
gamma	0, 1	✓	✓
lambda	1, 5	✓	✓
alpha	0, 1	✓	✓

a XGBoost: Extreme Gradient Boosting.

b A ✓ indicates applicability to the given task type.

For both CPH and XGBoost, input feature matrices were constructed using one-hot encoding for categorical variables. For variables with many categories (eg, country of birth and income bracket), only the 10 most frequent categories were one-hot encoded, and the remaining categories were grouped into an “other” category to reduce sparsity.

Missing values were handled as follows. For time-related variables, such as days since a measurement or diagnosis, missing values were imputed with zero under the assumption that missingness reflects the absence of a prior event or that no measurement had been recorded. For continuous variables such as laboratory values, missing entries were imputed using the median of the respective variable to reduce the influence of outliers while preserving representative values. For categorical variables, we imputed a dedicated “Missing” category. Because missingness in routine care data may itself carry predictive information, we additionally included binary missingness indicators for each variable. This allowed models to learn whether the presence or absence of a measurement (eg, a laboratory test) was informative, rather than forcing missing values to be treated as purely random noise. The imputation and indicator strategy was applied consistently across modeling frameworks to ensure comparability of feature matrices. In addition, tree-based models such as XGBoost can natively handle missing values by learning a default split direction for missing observations at each node, reducing reliance on complex imputation. We nevertheless applied a consistent imputation strategy across models to ensure comparability across modeling frameworks.

In addition, implementing multiple imputation by chained equations is computationally challenging in high-dimensional settings such as ours (>550 predictors, mixed variable types, and ~58,000 inclusion events). In preliminary attempts, multiple imputation by chained equations did not complete reliably due to memory and runtime constraints when including the full predictor set. Developing and validating a scalable multiple imputation pipeline for this linked dataset, including appropriate sensitivity analyses for nonrandom missingness, was outside the scope of this prediction-focused study. For transparency, we report the extent of missingness per variable group in Multimedia Appendix 1.

Model performance was evaluated primarily using the AUC metric, with 95% CIs provided. All metrics were computed using standard packages in R, including *survival*, *glmnet*, and *pROC*. In the second experiment, we determined via AUC which model performs best on the task of event prediction and then used the respective model in further experiments to quantify the added value of SDOH in the task of disease progression in risk patients.

To enhance the interpretability of the final ML models, we applied Shapley additive explanations (SHAP) to the selected models in the third experiment and beyond. SHAP values provide additive, locally accurate explanations that quantify both the direction and magnitude of each predictor’s contribution to an individual prediction [41]. For global feature importance, we summarized SHAP values using the mean absolute SHAP value per predictor across a held-out evaluation set. This metric reflects the average magnitude of a feature’s contribution to the model output, irrespective of direction, and enables a stable ranking of influential predictors. To support interpretability beyond ranking alone, we used a beeswarm plot to additionally examine the sign and distribution of SHAP values for key predictors in order to assess whether respective higher values were associated with increased or decreased predicted risk. To quantify the contribution of SDOH, SHAP values were further aggregated across predefined variable groups (medical vs social predictors), supporting interpretation of how these domains jointly influence predicted CVD and DM2 disease progression.

## Results

### Participant Characteristics

Table 2 presents baseline characteristics of the study population, consisting of 58,627 records from 48,687 unique individuals, of whom 6592 (13.5%) experienced a DM2 or CVD event during follow-up. Women comprised 53% (25,687/48,687) of the overall cohort, with a significantly higher proportion in the nonevent group (22,634/42,095, 54%) compared to the event group (2826/6592, 43%; *P*<.001). The mean age at inclusion was higher among individuals who experienced an event (59.68, SD 11.92 years) than among those who did not (53.55, SD 12.37 years; *P*<.001).

Age distributions also differed markedly between groups: individuals in the event group were more likely to be older, particularly aged 60‐70 years (28.87% vs 21.44%) and 70 years and older (21.95% vs 10.64%), whereas younger individuals (aged 30‐50 years) were more prevalent in the nonevent group. Among the most prevalent outcomes were codes T90 (25.02%), K75 (21.44%), and K89 (19.96%). The average time to event was 5.6 (SD 4.5) years across all outcomes.

Socioeconomic position differed significantly between groups. Individuals who experienced an event had lower household income indicators, including lower primary household income and lower standardized disposable income (both *P*<.001). Additionally, their households were smaller (mean household size 2.23, SD 1.12 vs 2.57, SD 1.64) and had fewer children (0.47, SD 0.88 vs 0.79, 1.08), with the children also being older at inclusion. These differences were all statistically significant (*P*<.001).

**Table 2.** Baseline characteristics of the study population, stratified by whether individuals experienced an incident CVD^a^ or DM2^b^ event during follow-up^c^.

Characteristic	Overall dataset	Experienced event	Did not experience event	Significance between groups (*P* value)
Overall statistics				
Number of rows	58,627	7842	50,785	—^d^
Number of people	48,687	6592	42,095	—
Women, n (%)	25,687 (52.76)	2826 (42.87)	22,634 (53.77)	<.001
Age at inclusion event (years), mean (SD)	54.37 (12.48)	59.68 (11.92)	53.55 (12.37)	<.001
Age at DM2 or CVD event (years), mean (SD)	—	65.27 (12.14)	—	—
Age category inclusion event (years), n (%)				
30‐40	6480 (13.31)	357 (5.42)	6116 (14.53)	—
40‐50	11,212 (23.03)	957 (14.51)	10,246 (24.34)	—
50‐60	14,158 (29.08)	1928 (29.25)	12,229 (29.05)	—
60‐70	10,921 (22.43)	1903 (28.87)	9025 (21.44)	—
>70	5916 (12.15)	1447 (21.95)	4479 (10.64)	—
Prevalence of DM2 or CVD event (top 5), n (%)				
Overall	7842 (13.38)	7429 (94.73)	—	—
K75^e^	1681 (2.87)	1681 (21.44)	—	—
K90^f^	1356 (2.31)	1356 (17.29)	—	—
K92.01^g^	865 (1.48)	865 (11.03)	—	—
T90^h^	1962 (3.35)	1962 (25.02)	—	—
K89^i^	1565 (2.67)	1565 (19.96)	—	—
Time to DM2 or CVD event (years; top 5), mean (SD)				
Overall	5.57 (4.48)	5.57 (4.48)	—	—
K75	5.42 (4.36)	5.42 (4.36)	—	—
K90	6.70 (4.48)	6.70 (4.48)	—	—
K92.01	5.84 (4.51)	5.84 (4.51)	—	—
T90	4.44 (4.28)	4.44 (4.28)	—	—
K89	6.13 (4.55)	6.13 (4.55)	—	—
Income statistics (in US $)^j^, mean (SD)				
Primary household income (labor+ self-employment+ capital)	200,524 (114,571)	1,193,103 (115,143)	201,834 (114,408)	<.001
Standardized spendable income (equivalized)	73,898 (26,398)	70,547 (23,825)	74,506 (26,794)	<.001
Household statistics, mean (SD)				
Number of people	2.53 (1.59)	2.23 (1.12)	2.57 (1.64)	<.001
Number of children	0.75 (1.06)	0.47 (0.88)	0.79 (1.08)	<.001
Youngest child at inclusion	14.02 (9.85)	16.62 (10.45)	12.77 (9.76)	<.001
Oldest child at inclusion	16.89 (9.20)	19.09 (9.65)	16.68 (9.13)	<.001
GP^k^ BMI (kg/m^2^)				
Prevalence of measurement, n (%)	16,140 (27.53)	2244 (28.62)	13,895 (27.36)	.02
Most recent value before inclusion, mean (SD)	30.26 (5.78)	30.07 (5.39)	30.30 (5.84)	.07
Highest value before inclusion, mean (SD)	30.92 (6.09)	30.86 (5.81)	30.92 (6.13)	.65
GP HbA_1c_ (mmol/mol)				
Prevalence of measurement, n (%)	1841 (3.14)	386 (4.92)	1452 (2.86)	<.001
Most recent value before inclusion, mean (SD)	41.39 (9.00)	44.59 (11.95)	40.54 (7.82)	<.001
Highest value before inclusion, mean (SD)	42.81 (10.87)	46.82 (13.74)	41.70 (7.82)	<.001
GP fasting glucose level (mmol/L)				
Prevalence of measurement, n (%)	27,496 (46.90)	3669 (46.79)	23,823 (46.91)	.85
Most recent value before inclusion, mean (SD)	5.89 (1.22)	6.30 (1.65)	5.83 (1.12)	<.001
Highest value before inclusion, mean (SD)	6.25 (1.75)	6.88 (2.46)	6.15 (1.59)	<.001
GP LDL^l^ cholesterol level (mmol/L)				
Prevalence of measurement, n (%)	25,901 (44.18)	3442 (43.89)	22,462 (44.23)	.59
Most recent value before inclusion, mean (SD)	3.45 (0.97)	3.47 (1.03)	3.45 (0.96)	.26
Highest value before inclusion, mean (SD)	3.86 (1.19)	3.96 (1.95)	3.84 (1.02)	<.001
GP HDL^m^ cholesterol level (mmol/L)				
Prevalence of measurement, n (%)	29,815 (49.32)	4075 (51.96)	24,839 (48.91)	<.001
Most recent value before inclusion, mean (SD)	1.34 (0.39)	1.28 (0.38)	1.35 (0.39)	<.001
Highest value before inclusion, mean (SD)	1.45 (0.96)	1.41 (1.43)	1.46 (0.86)	.03
GP triglycerides level (mmol/L)				
Prevalence of measurement, n (%)	28,012 (47.78)	3901 (49.74)	24,113 (47.48)	<.001
Most recent value before inclusion, mean (SD)	1.71 (1.32)	1.92 (1.48)	1.68 (1.29)	<.001
Highest value before inclusion, mean (SD)	2.11 (2.36)	2.38 (2.20)	2.07 (2.38)	<.001
GP total cholesterol level (mmol/L)				
Prevalence of measurement, n (%)	29,765 (50.77)	4190 (53.43)	25,575 (50.36)	<.001
Most recent value before inclusion, mean (SD)	5.52 (1.11)	5.57 (1.17)	5.51 (1.10)	.002
Highest value before inclusion, mean (SD)	6.02 (1.19)	6.17 (1.23)	6.00 (1.19)	<.001

a CVD: cardiovascular disease.

b DM2: type 2 diabetes.

c Event status refers to occurrence at any point during follow-up.

d Not applicable.

e International Classification of Primary Care code K75: myocardial infarction.

f International Classification of Primary Care code K90: cerebral infarction.

g International Classification of Primary Care code K92.01: peripheral artery disease.

h International Classification of Primary Care code T90: diabetes mellitus.

i International Classification of Primary Care code K89: transient ischemic attack.

j €1.00=US $1.15740658 (as of June 12, 2026).

k GP: general practitioner.

l LDL: low-density lipoprotein.

m HDL: high-density lipoprotein.

Biomedical profiles also differed substantially. Prior to inclusion, individuals in the event group had significantly worse metabolic profiles: higher mean hemoglobin A_1c_ levels (41.4, SD 9 vs 44.6, SD 12 mmol/mol), fasting glucose (6.30, SD 1.65 vs 5.83, SD 1.12 mmol/L), and triglycerides (1.92, SD 1.48 vs 1.68, SD 1.29 mmol/L), all with *P*<.001. LDL cholesterol levels were slightly lower in the event group, though the difference was small. BMI values showed only minor differences, with the most recent value slightly lower in the event group (30.1, SD 5.4 vs 30.3, SD 5.8 kg/m²; *P*=.07).

Overall, nearly all characteristics showed statistically significant differences between individuals who did and did not experience a CVD or DM2 event, highlighting the disparities between groups in socioeconomic, household, and clinical domains.

### Experiment 1: 10-Year CVD Prediction

The SCORE2 model achieved an AUC of 0.704 (95% CI 0.697‐0.711) on the full dataset and 0.697 (95% CI 0.680‐0.713) on the test set. The CPH model showed a significantly better performance (*P*<.001), reaching an AUC of 0.718 (95% CI 0.702‐0.733).

Among ML approaches, the binary random forest model achieved an AUC of 0.709 (95% CI 0.692‐0.724), performing comparably to SCORE2 but below CPH. XGBoost achieved the highest performance, with the survival model achieving an AUC of 0.727 (95% CI 0.711‐0.742) in the test set, statistically significantly outperforming the CPH model (*P*=.03). The binary XGBoost model achieved an AUC of 0.729 (95% CI 0.714‐0.745), which was not statistically different from the XGBoost survival model (*P*=.39). These findings indicate that ML-based approaches, such as XGBoost, can significantly improve risk discrimination for 10-year CVD compared to both regression and rule-based baselines, and also outperform a nonboosted tree ensemble baseline.

For the binary XGBoost model, we report the random search hyperparameters that yielded the highest performance in the 10-year CVD event prediction task. The best-performing configuration used a maximum tree depth (*max_depth*) of 9, a learning rate (*η*) of 0.1, a subsample ratio of 0.7, a column subsample ratio per tree (*colsample_bytree*) of 0.7, a minimum child weight of 1, and regularization parameters set as follows: *γ*=1, λ=1, and *α*=1.

### Experiment 2: Overall Event Prediction

The CPH model achieved an AUC of 0.704 (95% CI 0.691‐0.717) in predicting time-to-event for the combined outcome of DM2 and CVD, illustrating its strength in handling censored survival data and modeling the temporal aspect of risk. The XGBoost survival model yielded a higher AUC of 0.715 (95% CI 0.701‐0.728), significantly outperforming CPH (*P*<.001), indicating that the survival-based boosting framework was able to capture additional predictive signal in this high-dimensional dataset.

The random forest model achieved an AUC of 0.705 (95% CI 0.691‐0.718), performing similarly to CPH (*P*=.81), but significantly below both XGBoost variants (both *P*<.001)

When reformulated as a binary classification task, predicting whether an event occurs at any point during the study period rather than modeling its timing, the XGBoost model significantly outperformed both survival models, achieving an AUC of 0.719 (95% CI 0.706‐0.733), although the absolute difference between the XGBoost variants was modest. This highlights the advantage of boosted tree-based ML methods in high-dimensional, nonlinear feature spaces when the outcome is event occurrence, rather than precise event timing.

As our comparison focuses on the discriminative ability of our models, how well each model stratifies risk between those who do and do not experience an event, our findings illustrate that while CPH remains robust and interpretable for time-to-event analysis, binary XGBoost offers superior performance for overall event prediction in complex datasets. Based on this, we proceeded with the binary XGBoost model in subsequent experiments, including our analysis of the added value of SDOH. This model was trained using the best-performing hyperparameters identified in our earlier random search.

### Experiment 3: Time-Bound Prediction Tasks (5- and 10-Year Event Prediction)

The 5-year overall event model achieved an AUC of 0.738 (95% CI 0.721‐0.755), significantly outperforming the 5-year CVD model (AUC 0.722, 95% CI 0.702‐0.743), while the difference was with the 5-year DM2 model (AUC 0.786, 95% CI 0.758‐0.814). The 5-year DM2 model performed significantly better than the CVD 5-year model.

For the 10-year window, the overall model achieved an AUC of 0.722 (95% CI 0.708‐0.736), which is significantly lower than both the 10-year CVD model (AUC 0.729, 95% CI 0.714‐0.745) and the 10-year DM2 model (AUC 0.760, 95% CI 0.735‐0.785). The differences between the CVD and DM2 models could be explained by the wider definition of CVD, which constitutes 6 of the 7 total event ICPC.

### Experiment 4: Contribution of SDOH

For the last set of experiments, we selected the 5-year overall binary XGBoost model to evaluate the added predictive value of biomedical risk factors versus SDOH. This model was chosen because it achieved strong performance and represents an interpretable and clinically relevant prediction task. To reduce multicollinearity and simplify interpretation, we tested a feature set excluding 2 highly correlated income variables (standardized disposable household income and gross household income) and replacing floored age with a single continuous age feature (days since date of birth). This reduced set slightly improved performance in the 5-year overall event model (AUC 0.734 vs 0.739; *P*=.02), with minimal effects on other models. We therefore adopted this reduced set for our main SDOH analysis.

Table 3 presents the predictive performance of models trained on different subsets of features to evaluate the added value of SDOH. The combined 5-year binary model from the previous experiment achieved an AUC of 0.738 (95% CI 0.721‐0.755). The model using only biomedical risk factors performed slightly lower, with an AUC of 0.728 (95% CI 0.711‐0.746), although the difference was statistically significant (*P*=.01), indicating that the inclusion of social determinants provides incremental improvement in the task of predicting disease onset in patients with elevated risk.

In contrast, the model trained exclusively on social determinants achieved an AUC of 0.691 (95% CI 0.683‐0.709), significantly lower than the medical-only model (*P*<.001), but still showing a meaningful predictive signal. A minimal baseline model containing only inclusion code and age performed worst, with an AUC of 0.659 (95% CI 0.641‐0.678), and was significantly outperformed by all other models.

To explore the impact of the most informative features, we also evaluated models using only the top 10 medical and/or social predictors, determined by mean absolute SHAP values in the overall 5-year binary model. The top 10 biomedical risk factors alone achieved an AUC of 0.729 (95% CI 0.712‐0.746), while the top 10 social determinants only reached 0.687 (95% CI 0.668‐0.705).

**Table 3.** XGBoost^a^ performance on different contexts relating to subsets of features.

XGBoost model	AUC^b^ (95% CI)
Overall 5-year binary model	0.738 (0.721‐0.755)
Only biomedical risk factors	0.728 (0.711‐0.746)
Only SDOH^c^	0.691 (0.683‐0.709)
Only inclusion code and age	0.659 (0.641‐0.678)
Only the top 10 biomedical risk factors	0.729 (0.712‐0.746)
Only the top 10 social determinants of health	0.687 (0.668‐0.705)
Top 10 biomedical + top 10 social determinants combined	0.738 (0.721‐0.755)

a XGBoost: Extreme Gradient Boosting.

b AUC: area under the receiver operating characteristic curve.

c SDOH: social determinants of health.

Notably, combining the top 10 biomedical and top 10 social predictors restored performance to the level of the full combined model. This finding suggests that the added predictive value of SDOH is not driven by a large number of weak social predictors, but can be captured by a small subset of highly informative social features that complement biomedical risk factors.

**Figure 1.**
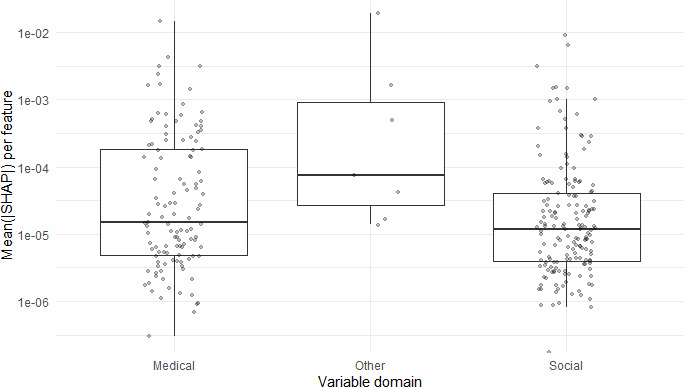
Boxplot of feature importance by variable domain, showing spread in mean absolute Shapley additive explanations values of the predictors in the 5-year Extreme Gradient Boosting model. SHAP: Shapley additive explanations.

To further interpret the role of biomedical and social predictors in the final 5-year binary XGBoost model, we analyzed SHAP values at both the global and individual levels. First, we summarized feature contributions by computing the mean absolute SHAP value per predictor and aggregating these values across predefined feature groups (biomedical, SDOH, and baseline characteristics), which we visualized in a box plot in Figure 1 below.

Biomedical predictors have substantially higher per-feature importance, with a higher median SHAP magnitude and a broader upper tail compared to SDOH. In contrast, SDOH features show lower average contributions per feature but still include a subset of variables with relatively large SHAP values, indicating that a small number of social predictors contribute meaningfully to risk discrimination. This pattern aligns with the model performance findings earlier in this experiment, where SDOH provided incremental predictive value when combined with biomedical determinants, despite being weaker as a stand-alone feature set. This is consistent with the observation that combining merely the top 10 biomedical and top 10 social predictors recovered the full model performance, suggesting that SDOH contribute primarily through a relatively small set of highly informative variables.

**Figure 2.**
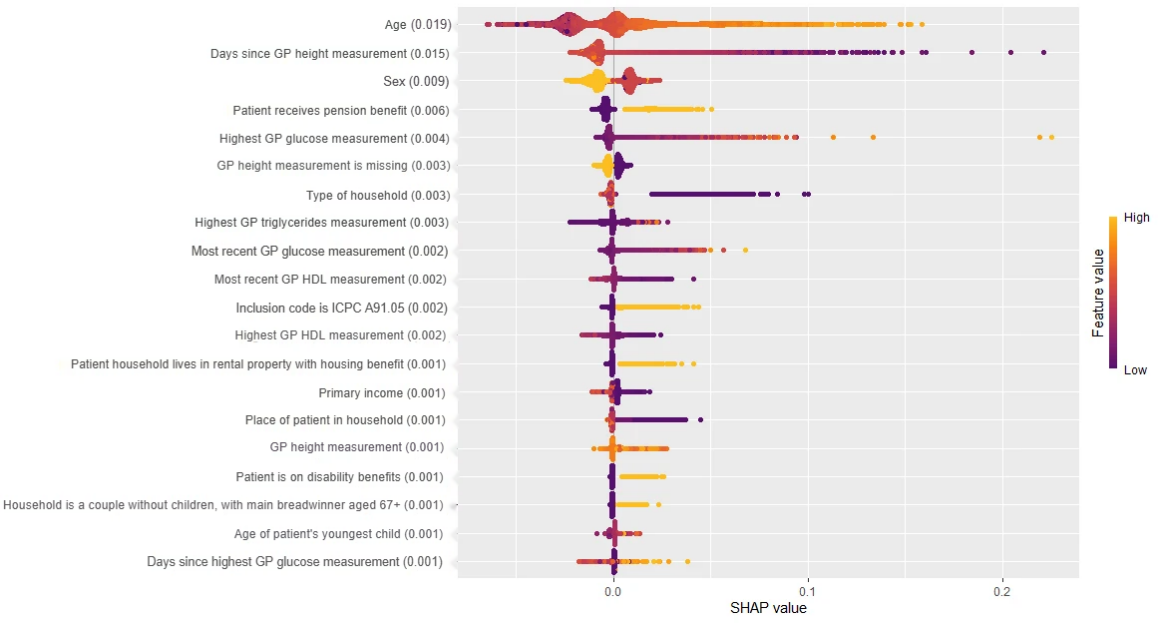
Beeswarm plot of Shapley additive explanations values among the top 20 predictors with the highest mean absolute Shapley additive explanations value within the 5-year Extreme Gradient Boosting event prediction model. The respective mean absolute Shapley additive explanations values are denoted in parentheses. GP: general practitioner; HDL: high-density lipoprotein; ICPC: International Classification of Primary Care; SHAP: Shapley additive explanations.

While the domain-level SHAP distributions summarize the relative contribution of biomedical and social predictors, they do not indicate which specific variables drive model predictions or whether their effects are associated with increased or decreased risk. To address this, Figure 2 presents a SHAP beeswarm plot of the top 20 predictors ranked by mean absolute SHAP value. Each point represents an inclusion event, with the SHAP value on the *x*-axis indicating the direction and magnitude of a feature’s contribution to the predicted risk of CVD or DM2 disease progression. Point color reflects the feature value, with higher values shown in lighter colors.

Age is the most influential predictor, with higher age consistently associated with increased predicted risk. Sex also contributed meaningfully, with male sex generally shifting predictions toward higher risk.

Several features related to clinical measurement patterns emerged as influential. In particular, lower values of “days since GP height measurement” were associated with increased predicted risk, suggesting that more recent clinical measurement activity coincided with higher risk profiles. Consistent with this, a missingness indicator for height measurement also contributed to predictions, indicating that the presence or absence of routine measurements carried predictive signal beyond the measured value itself.

Multiple socioeconomic and household-level variables appeared among the most influential predictors. Receipt of pension benefits was associated with increased predicted risk, likely reflecting its strong correlation with older age and retirement status. Housing-related variables also showed clear patterns: living in rental housing with housing benefit was associated with higher predicted risk, consistent with socioeconomic disadvantage. Similarly, disability benefit status shifted predictions toward higher risk, suggesting that disability and work limitations capture an additional vulnerability signal not fully explained by biomedical measurements alone.

Finally, household composition variables contributed to risk prediction in a more heterogeneous way. The categorical “type of household” feature showed a distinct pattern in which the “unknown” category was associated with increased risk in combination with other predictors. The other household types did not exhibit much predictive power, further highlighting the importance of this type. This suggests that missing or unclassified household registration may itself act as a proxy for instability or unmeasured social complexity, which the model uses when estimating CVD and DM2 risk.

Together, these patterns reinforce the findings described above that the added value of SDOH is driven primarily by a limited set of informative social variables that complement biomedical variables, rather than by meaningful contributions across a large number of weak predictors.

### Sensitivity Analysis: Single-Event Versus Multievent Dataset Construction

To assess whether the multievent dataset structure influenced predictive performance through within-patient dependence, we conducted sensitivity analyses using alternative dataset constructions in which only a single inclusion event per patient was retained. Specifically, we evaluated XGBoost models trained on datasets containing either the first or last inclusion event per individual and compared their performance with the primary multievent model. The task at hand was 5-year event prediction.

Using a single inclusion event per patient, the XGBoost model trained on first inclusion events achieved an AUC of 0.746 (95% CI 0.727‐0.764) for 5-year overall event prediction, while the model trained on last inclusion events achieved a slightly lower AUC of 0.735 (95% CI 0.717‐0.753). This difference was not statistically significant (*P*=.41), indicating comparable discrimination between first- and last-event representations.

To further distinguish the effects of dataset structure from model behavior, we performed cross-evaluation analyses. When the model trained on first inclusion events was evaluated on the multievent test set, it achieved an AUC of 0.738, which was identical to the performance of the model trained and evaluated on the multievent dataset (AUC 0.738; *P*=.71). Furthermore, when the model trained on the multievent dataset was evaluated on the first-event test set, it again achieved the same AUC as the first-event-trained model evaluated on that test set (both AUC 0.746; *P*=.99)

These findings indicate that observed differences in performance between single-event and multievent analyses are driven primarily by differences in test set composition rather than by model overfitting or inflation due to repeated observations per patient. Overall, model discrimination and comparative conclusions were stable across dataset constructions, supporting the robustness of the primary multievent modeling approach, which is maintained in the other experiments.

## Discussion

### Principal Results

This study demonstrates that adding SDOH on top of biomedical data significantly improves the prediction of disease progression to CVD and DM2. In our best-performing model, a binary XGBoost classifier predicting 5-year progression to either DM2 or CVD, several socioeconomic variables (including income and social benefit indicators) ranked among the top predictors, performing equally or outperforming traditional biomedical features such as laboratory values and diagnosis codes. Although models trained solely on social data underperformed compared to those using only biomedical data, the integration of both domains produced superior results, confirming the complementary value of SDOH, which we directly quantified using the XGBoost gain metric.

In the construction of our dataset, we used routine care data actively recorded by health care professionals within ELAN-affiliated practices and hospitals. This allowed us to define a clinically grounded high-risk cohort based on structured diagnosis and prescription records, reflecting how risk is typically identified in real-world primary care settings. We first compared the performance of SCORE2, CPH, and XGBoost models on the task of predicting 10-year progression to CVD. Although smoking status was unavailable, the model achieved performance comparable to the SCORE2 ELAN validation by Kist et al [17], supporting its use as a reference baseline.

Across experiments, we observed differences in performance between modeling frameworks. For overall event prediction, CPH, survival XGBoost, and binary XGBoost were all evaluated using AUC on the binary event end point. In this setting, survival XGBoost achieved slightly higher discrimination than CPH, while binary XGBoost achieved the highest AUC overall. This suggests that, in this high-dimensional dataset, boosted tree models can capture predictive signal beyond proportional hazards regression for clinically actionable risk stratification. Random forest provided a competitive tree-based baseline but did not match the performance of boosted trees, indicating that the observed gains were not solely attributable to nonlinear modeling but also to the sequential boosting framework.

The added value of SDOH was further supported by SHAP-based interpretability analyses. Biomedical predictors showed higher average contribution on a per-feature basis, but a subset of social predictors consistently ranked among the most influential features in the full model. Notably, combining only the top 10 biomedical and top 10 social predictors recovered the full model performance, suggesting that the predictive contribution of SDOH is driven primarily by a limited number of highly informative social variables rather than diffuse contributions across many weak predictors. Together, these findings support the inclusion of SDOH in modeling CVD and DM2 risk and suggest that a compact set of social and biomedical indicators may be sufficient to achieve strong discrimination in routine-care prediction settings.

### Comparison With Prior Work

Our findings align with growing evidence that integrating SDOH significantly enhances the prediction of CVD and DM2 outcomes. Prior studies, such as those by Xia et al [42] and Cooper et al [43], have demonstrated improved model performance when including socioeconomic or area-level indicators. However, many of these studies used smaller, survey-based cohorts or relied on aggregated SDOH data, such as area-level income or education, which limits precision and generalizability.

More recent studies using larger datasets and advanced ML methods further underscore the added predictive value of SDOH. For example, Teshale et al [44] incorporated self-reported SDOH alongside traditional clinical risk factors in ML and deep learning models trained on a cohort of over 12,000 older adults (aged ≥70 years) from Australia and the United States. Their findings showed that SDOH, such as social network, living situation, and education, were as predictive as, or more predictive than, clinical variables, particularly among women. Similarly, Howell et al [45] demonstrated that including self-reported SDOH modestly improved the predictive performance of diabetes risk models in a cohort of nearly 10,000 American adults aged 45 years and older, all without DM2 at baseline. Socioeconomic and behavioral variables such as health insurance status, income, and health literacy contributed meaningful information. Our study differs in scale from these studies, with a larger dataset, but also in the use of objectively registered SDOH, rather than self-reported, enhancing data reliability and creating a more comprehensive view of the added value of SDOH in estimating the risk of developing CVD and DM2.

Segar et al [46] also showed that SDOH can enhance prediction in large-scale clinical settings. In their study, which used a large training set of 123,000 American patients with heart failure, incorporating SDOH data into ML models significantly improved the prediction of in-hospital mortality. However, again, this study, like many others, relied on aggregated, area-based social measures rather than individual-level data.

A recent non-Western example is the study by Hu et al [47], which used ML to predict DM2 incidence among over 26,000 adults from Fujian province in China. Their model, based on a feature set of 18 self-reported variables, including age, BMI, education, income, marital status, smoking status, and self-rated health, achieved an AUC value of 0.723. The study found that SDOH, such as rural residence, low income, and low education levels, were strongly associated with increased risk of developing DM2, underscoring the relevance of social factors beyond Western health care contexts.

It is important to note that most of the existing studies were conducted in the United States, where the health care system differs substantially from that of the Netherlands in terms of access, financing, and the role of socioeconomic factors. The Dutch health care system offers universal coverage and more standardized access to primary care, potentially altering how social determinants affect health outcomes. Nevertheless, European research supports the broader relevance of SDOH even within universal care systems. For example, analysis of data from the Survey of Health Ageing and Retirement in Europe study (~14,000 participants aged 50‐75 years in Belgium and France) showed that socioeconomic conditions, including education and social network measures, independently predicted mortality and major cardiovascular events [48]. These findings reinforce the idea that SDOH remain important predictors even when basic health care access is guaranteed, a conclusion that is consistent with the results of this study.

We further distinguish our approach by comparing multiple predictive models, including ML (XGBoost) techniques, trained with and without SDOH. This systematic comparison allows us to directly quantify the added value of social data beyond medical predictors alone, a dimension that is often overlooked or only partially explored in prior research.

Moreover, by applying SHAP in the third experiment and beyond, we provide interpretable insights into both global and individual-level contributions of biomedical and social predictors. We furthermore show directionality and domain-level aggregation plots, creating a basis for further research and clinical prioritization.

### Strengths and Limitations

This study benefits from several methodological and data-related strengths. Most notably, we constructed a large, real-world dataset comprising more than 58,000 inclusion events from nearly 49,000 individuals. By linking primary care, secondary care, and CBS data at the individual level, we were able to include a wide range of SDOH, such as gross and disposable income, household structure, and migration background. Crucially, unlike previous studies that rely on self-reported (social) data, our social variables are drawn from mandatory national registries, including municipal population databases that include all legal Dutch residents. This eliminates self-report bias, improves completeness, and allows for consistency over time through annually updated information, independent of survey timing or participant response, achieving a level of precision and coverage not previously demonstrated in related literature.

By comparing models trained with and without SDOH, we provide both robust predictive performance and interpretable insights into the relative contribution of biomedical and social factors. The inclusion event-based design ensures alignment with actual clinical decision points, reinforcing the practical utility of our predictive models.

The scale and objectivity of our data allow for a comprehensive and nuanced quantification of the individual contribution of social factors in predicting CVD and DM2 outcomes. Moreover, the breadth of SDOH included in our study, many of which are unavailable or sparsely measured in prior studies, allows for more comprehensive modeling of social conditions affecting CVD and DM2 risk than prior studies that do not make use of such an extensive feature set. The broader coverage helps to isolate unique effects of individual social factors, improves the accuracy in selecting the most relevant predictors, and provides a stronger foundation for investigating potential causal relationships in future research.

A further key strength of our study lies in its clinically grounded definition of CVD and DM2 risk. In consultation with clinical experts, we identified high-risk individuals using structured diagnostic records, closely mirroring real-world identification in primary care. While this approach may limit strict comparability to studies based solely on metabolic syndrome criteria, it enhances practical relevance and replicability across real-world health care settings.

Despite these strengths, several limitations should be acknowledged to contextualize the findings of this study. For example, in the construction of our dataset, we were limited to routine care data that were actively recorded by health care professionals within ELAN-affiliated practices and hospitals. This means that we only have information from points in time when patients interact with the health care system, for example, through GP visits or hospital consultations. As a result, we lack visibility into the health of patients during periods when they do not interact with the health care system. This introduces a substantial systematic bias toward people who are more likely to seek care and have risk factors recorded. Furthermore, since our dataset is structured around inclusion events rather than unique individuals, patients with higher health care involvement can contribute multiple entries to training data. This may further amplify the bias by overrepresenting those who have more frequent interactions, skewing model learning toward more medically active subpopulations, and possibly leaning toward more severe cases. We tried to mitigate potential data leakage by not allowing the same patients to appear in both the training and test sets; however, this does not fully address the bias introduced by the overrepresentation of highly engaged patients. This bias can have several important implications. First, it may lead to an underrepresentation of people who have difficulties accessing medical care, including those with a lower socioeconomic position. These same individuals may be at elevated risk due to unmeasured or unaddressed medical and social vulnerabilities. Moreover, these individuals may seek care only when conditions become more severe, meaning the available data on these patients may disproportionately reflect more advanced disease stages.

On the other hand, patients who frequently interact with the health care system may appear to have more complete risk profiles, potentially increasing the predictive power of features such as laboratory values or diagnosis codes that are only available when recorded. This could create overfitting to data patterns that may not generalize well to less engaged patient populations. Future research could help quantify and address this bias by comparing model performance across specific, clinically relevant patient subgroups.

This limitation demonstrates a larger challenge in retrospective clinical data research and is difficult to fully mitigate. Addressing it would require more continuous population-based data collection, such as through wearable devices or patient-reported outcomes. When such data are not available, future work could explore model performance across population subgroups, for example, stratified by ethnicity or practice location, to better understand disparities in prediction performance among different populations. However, in this study, we show that it is feasible to determine the risk of disease progression when evaluating inclusion events.

Cardiovascular mortality was deliberately not included as a separate outcome in this study because the primary objective of our modeling framework is to support early, clinically actionable prevention rather than late-stage fatality prediction. We acknowledge, however, that death occurring before a recorded CVD diagnosis represents a competing event that may prevent observation of the nonfatal outcomes studied here. Cause-specific cardiovascular mortality was not modeled separately, and competing-risk modeling was outside the scope of the current work. To reduce the likelihood that mortality dominates follow-up and to maintain focus on prevention-relevant risk stratification, we restricted cohort inclusion to individuals aged ≤70 years at entry. Future work could extend this framework by incorporating mortality outcomes and competing-risk methods when appropriate linkage and outcome registration are available.

In addition, our SHAP analyses indicated that variables reflecting measurement patterns, especially the presence of a recorded height measurement and the number of days since the last height measurement, were among the most influential predictors. These features likely capture aspects of health care use and clinical monitoring intensity (eg, height being recorded as part of weight management or cardiometabolic screening) rather than solely reflecting underlying physiology. Consequently, the model may partly learn patterns of clinical attention (ie, who is monitored more closely) rather than only underlying disease risk. This could inflate predictive performance in routine-care data and reduce generalizability to individuals with fewer recorded measurements or less frequent health care contact.

Due to the large number of linked variables used in our analysis and our dataset, we are able to offer a comprehensive view of which social and biomedical risk factors are impactful in predicting the onset of CVD and DM2. However, despite this extensive scope, certain variables known to influence CVD and DM2 risk were not available. Most notably, we were unable to include smoking status, substance abuse, physical activity, and dietary habits, all of which are established lifestyle factors associated with CVD and DM2. These factors are typically not captured in ELAN routine care data in a structured format, and unstructured data were also unavailable for this study. Although our models compensate for some of the missing factors through related variables, such as income as a potential (although shallow) indicator of socioeconomic position, the absence of these factors might harm the predictive accuracy of the models. Future research could aim to integrate lifestyle and behavioral data, that is, through free-text clinical notes [49], to further enhance model performance and understanding of CVD and DM2 risk. Nevertheless, our models achieved robust predictive performance comparable to or exceeding that reported in existing literature, even without direct lifestyle and behavioral data.

An additional consideration is that several high-ranking SDOH variables are inherently correlated with age and life stage. For example, receipt of pension benefits, household indicators related to retirement age, and the age of children are strongly age-dependent and may partly reflect the dominant role of age in CVD and DM2 risk prediction. However, other influential SDOH features, such as disability benefits and housing-related indicators (eg, rental housing and housing benefit), are less directly tied to age and likely capture complementary vulnerability signals.

We compared both survival-based and binary modeling formulations. In our experiments, survival XGBoost achieved discrimination comparable to, and slightly higher than, CPH when evaluated on a common binary event end point, but absolute performance differences between survival and binary formulations were modest. This suggests that, in high-dimensional routine-care data, direct optimization of binary event prediction may be advantageous for fixed-horizon clinical risk stratification, even when time-to-event information is available.

Importantly, although CPH and survival XGBoost were trained using time-to-event objectives, our primary evaluation focused on discrimination for clinically actionable binary end points (eg, event occurrence within 5 or 10 years), rather than survival-specific metrics such as time-dependent AUC or concordance. As a result, the reported AUC values reflect performance for fixed-horizon risk stratification and may not fully capture the potential advantages of survival models for modeling event timing. Future work could evaluate survival models using survival-specific metrics and calibration at clinically relevant time horizons.

At the same time, this study should be interpreted as a model development and internal validation study within a single regional health care infrastructure. Although our evaluation was performed out of sample within this setting and patient-level splitting prevents direct leakage, model performance and calibration may not fully generalize to other regions or health care systems with different population characteristics, care pathways, coding practices, or availability of SDOH variables. Importantly, comparable datasets with similarly extensive linked SDOH are currently uncommon, which makes immediate external validation challenging. Nevertheless, external validation and recalibration in independent cohorts remain essential steps before clinical deployment.

### Clinical Implications

While our dataset contains a presenting bias, data being available only when patients interact with the health care system, this limitation is also a key opportunity for clinical application. Predictive models like the ones developed in this study are most useful at moments when patients present themselves. By making use of routinely collected data from primary care and hospitals, enriched with SDOH, our models can assist health care providers in identifying patients at elevated risk for CVD and DM2 disease progression in real time.

Such models could support primary care workflows, where time constraints limit comprehensive risk assessment, if externally validated. Here, a risk prediction tool integrated into electronic health record systems could flag high-risk individuals at the point of care, possibly enabling earlier interventions. Notably, the added value of SDOH, measured via the clinically interpretable SHAP method, supports the idea that socially vulnerable individuals may benefit from more tailored approaches. By capturing these signals, predictive models can also help clinicians identify patients who may not present with abnormal laboratory values but who are nonetheless at elevated long-term risk. Given that our approach was rooted in real-world clinical data and is scalable to similar infrastructures, it provides a foundation for integrating these predictive analytics into routine care, contributing to more data-informed health care.

However, the integration of SDOH into existing clinical systems remains a practical challenge. Many health care systems do not currently capture detailed social data in a structured or standardized format, and the inclusion of such data may require changes in infrastructure and an ethical discussion. For this reason, biomedical-only models, like the one evaluated in this study, can still offer meaningful improvements over existing practices and may serve as an intermediate or fallback solution when SDOH data are unavailable. Although these models perform slightly worse than combined models, they remain clinically valuable and are more readily implementable in most care settings. Future research could explore how such models can be validated and implemented, including pilot implementations and usability studies with clinicians.

### Conclusions

This study demonstrates the added value of incorporating SDOH into predictive models for progression to CVD or DM2. Using a clinically grounded, high-risk cohort identified through structured diagnosis records in ELAN-affiliated practices and linking these to individual-level sociodemographic data from CBS, we created a uniquely comprehensive dataset to examine the interplay between medical and social risk factors. While biomedical features remained dominant predictors, social characteristics substantially enhanced model performance when combined with clinical data, underscoring their value as complementary signals in personalized risk prediction.

Using XGBoost in a high-dimensional setting enabled robust prediction across hundreds of linked predictors, while SHAP-based interpretability analyses provided transparent insight into the relative contribution of biomedical and social factors. Notably, the combined predictive value of SDOH was largely captured by a limited subset of highly informative social variables, as reflected by the strong performance of models trained on the top-ranked medical and social predictors.

Although the integration of SDOH into clinical practice remains logistically challenging due to infrastructure and data standardization and ethical issues, our findings suggest that predictive models using routine biomedical data alone still provide meaningful insights and could serve as an effective starting point for implementation. Altogether, our study highlights the potential of socially informed risk models to support personalized prevention strategies and more equitable care, and offers a replicable framework for future applications in real-world clinical settings.

## Supplementary material

10.2196/80377 Multimedia Appendix 1 Shapley additive explanations–based feature importance rankings for the 5-year type 2 diabetes and cardiovascular disease Extreme Gradient Boosting model, including variable domain (medical, social, or other), feature type, and proportion of nonmissing values.

## Abbreviations

AUC: area under the receiver operating characteristic curve
CBS: Statistics Netherlands (*Centraal Bureau voor de Statistiek*)
CPH: Cox proportional hazards
CVD: cardiovascular disease
DM2: type 2 diabetes
ELAN: Extramural Leiden University Medical Center Academic Network
GP: general practitioner
HDL: high-density lipoprotein
ICPC: International Classification of Primary Care
LASSO: Least Absolute Shrinkage and Selection Operator
LDL: low-density lipoprotein
LUMC: Leiden University Medical Center
MetS: metabolic syndrome
ML: machine learning
SCORE: Systematic Coronary Risk Evaluation
SCORE2: Systematic Coronary Risk Evaluation 2
SDOH: social determinants of health
SHAP: Shapley additive explanations
WMO: Medical Research Involving Human Subjects Act
XGBoost: Extreme Gradient Boosting
