# Supplementary material for: Quantifying the Predictive Power of Social Determinants of Health in Cardiovascular Disease and Type 2 Diabetes Progression Using XGBoost: Retrospective Cohort Study
Source: JMIR Med Inform. 2026 Jul 9;14:e80377. doi: 10.2196/80377 (PMC13349322; doi:10.2196/80377)
Supplement: Multimedia Appendix 1 [file medinform-v14-e80377-s001.docx]

**Quantifying the Predictive Power of Social Determinants of Health in Cardiovascular Disease and Type 2 Diabetes Progression Using XGBoost**

**Supplementary Material**

**Feature Importance**

The results in Table S1 are based on the XGBoost model developed to predict the occurrence of a DM2 or CVD diagnosis within 5 years of dataset inclusion. The model was trained on 46.895 entries, containing 37.668 unique individuals. Each inclusion event was represented by 658 predictor variables spanning biomedical and social domains.

Table S1 presents the global feature importance derived from SHAP (SHapley Additive exPlanations) values. For each feature, we report:

- Rank: the position of the feature when ordered by mean absolute SHAP value (highest to lowest).
- Feature: the name of the predictor variable.
- Group: the predefined domain of the feature (Medical, Social, or Other).
- Type: whether the feature is numerical or categorical.
- Mean absolute SHAP value: the average absolute SHAP value across the evaluation set. This reflects the average magnitude of the feature’s contribution to the model output, irrespective of direction. Higher values indicate greater influence on predicted risk.
- Proportion not missing: the proportion of inclusion events for which the feature had a non-missing value. This provides insight into the availability and measurement frequency of each predictor in the dataset.

SHAP values quantify each feature’s contribution to individual predictions and allow for a more interpretable assessment of feature influence. By ranking features using mean absolute SHAP values, we identify predictors that most strongly contribute to risk discrimination in the final model.

The Group variable distinguishes biomedical determinants (derived from GP and hospital records), social determinants of health (derived from Statistics Netherlands), and baseline/meta characteristics (e.g., age and inclusion-related variables). Reporting both SHAP importance and data availability allows for interpretation of whether highly influential predictors are widely measured across the cohort or reflect more selective clinical recording patterns.

In this table, “Hospital 1” refers to HagaZiekenhuis, while “Hospital 2” refers to the Haaglanden Medisch Centrum, both in The Hague. “Combined hospital” refers to combining the respective measures of both hospitals, where we take either the most recent or the highest value among the values of the both hospitals among the individuals in our dataset.

**Table S1.** The top 100 features in the 5 year event prediction XGBoost model, ordered by the SHAP metric in descending order.

| Rank | Feature | Group | Type | Mean Absolute SHAP Value | Proportion of Column  not Missing (%) |
| --- | --- | --- | --- | --- | --- |
| 1 | Age | Other | Numerical | 1.90 * 10^-2^ | 99.3 |
| 2 | Days since GP Height Measurement | Medical | Numerical | 1.45 * 10^-2^ | 51.74 |
| 3 | Sex | Social | Categorical | 9.14 * 10^-3^ | 99.3 |
| 4 | Patient Receives Pension Benefit | Social | Categorical | 6.45 * 10^-3^ | 61.75 |
| 5 | Highest GP Glucose Measurement | Medical | Numerical | 4.20 * 10^-3^ | 46.9 |
| 6 | GP Height Measurement is Missing | Medical | Categorical | 3.17 * 10^-3^ | 100 |
| 7 | Type of Household | Social | Categorical | 3.14 * 10^-3^ | 95.76 |
| 8 | Highest GP Triglycerides Measurement | Medical | Numerical | 3.10 * 10^-3^ | 47.87 |
| 9 | Most Recent GP Glucose Measurement | Medical | Numerical | 2.41 * 10^-3^ | 46.9 |
| 10 | Most Recent GP HDL Measurement | Medical | Numerical | 1.70 * 10^-3^ | 49.32 |
| 11 | Inclusion Code is ICPC A91.05 | Other | Categorical | 1.64 * 10^-3^ | 100 |
| 12 | Highest GP HDL Measurement | Medical | Numerical | 1.60 * 10^-3^ | 49.38 |
| 13 | Patient Household Lives in Rental Property with Housing Benefit | Social | Categorical | 1.50 * 10^-3^ | 61.75 |
| 14 | Primary Income | Social | Numerical | 1.46 * 10^-3^ | 61.75 |
| 15 | Place of Patient in Household | Social | Categorical | 1.46 * 10^-3^ | 95.76 |
| 16 | GP Height Measurement | Medical | Numerical | 1.41 * 10^-3^ | 51.67 |
| 17 | Patieint is on Disability Benefits | Social | Categorical | 1.01 * 10^-3^ | 61.75 |
| 18 | Household is a Couple without Children, with Main Breadwinner Aged 67+ | Social | Categorical | 9.92 * 10^-4^ | 61.75 |
| 19 | Age of Patient's Youngest Child | Social | Numerical | 9.46 * 10^-4^ | 39.44 |
| 20 | Days since Highest GP Glucose Measurement | Medical | Numerical | 8.41 * 10^-4^ | 50.86 |
| 21 | Spendable Income | Social | Numerical | 6.75 * 10^-4^ | 61.67 |
| 22 | Days since Most Recent GP Weight Measurement | Medical | Numerical | 6.37 * 10^-4^ | 30.01 |
| 23 | Days since Highest GP Weight Measurement | Medical | Numerical | 6.17 * 10^-4^ | 29.91 |
| 24 | Standardised Spendable Income | Social | Numerical | 6.12 * 10^-4^ | 61.75 |
| 25 | Most Recent GP Triglycerides Measurement | Medical | Numerical | 6.01 * 10^-4^ | 47.78 |
| 26 | Highest GP Cholesterol Measurement | Medical | Numerical | 5.90 * 10^-4^ | 50.86 |
| 27 | Household is a Single Man, Aged 67+ | Social | Categorical | 5.10 * 10^-4^ | 61.75 |
| 28 | Days since Most Recent GP Glucose Measurement | Medical | Numerical | 5.04 * 10^-4^ | 46.98 |
| 29 | Inclusion Code is ICPC K85 | Other | Categorical | 4.91 * 10^-4^ | 100 |
| 30 | Most Recent GP LDL Measurement | Medical | Numerical | 4.77 * 10^-4^ | 44.18 |
| 31 | Days since Highest GP LDL Measurement | Medical | Numerical | 4.77 * 10^-4^ | 44.47 |
| 32 | Days since Highest GP Glucose Measurement | Medical | Numerical | 4.71 * 10^-4^ | 46.91 |
| 33 | Days since Highest GP HDL Measurement | Medical | Numerical | 4.07 * 10^-4^ | 49.39 |
| 34 | Highest GP BMI Measurement | Medical | Numerical | 4.07 * 10^-4^ | 27.49 |
| 35 | Most Recent GP HbA1c Measurement | Medical | Numerical | 3.95 * 10^-4^ | 3.14 |
| 36 | Household is a Couple, Both Aged 67+ | Social | Categorical | 3.80 * 10^-4^ | 61.75 |
| 37 | Degree of Benefit Dependency of Private Households in the Last Four Years, Category is Not One of Top 10 Most Frequent | Social | Categorical | 3.78 * 10^-4^ | 61.75 |
| 38 | Highest GP LDL Measurement | Medical | Numerical | 3.55 * 10^-4^ | 44.47 |
| 39 | Highest GP HbA1c Measurement | Medical | Numerical | 3.31 * 10^-4^ | 3.24 |
| 40 | Most Recent GP BMI Measurement | Medical | Numerical | 3.05 * 10^-4^ | 27.53 |
| 41 | 18th Percentile Group of Gross Income of Private Households | Social | Categorical | 2.97 * 10^-4^ | 61.75 |
| 42 | Taxable Income | Social | Numerical | 2.86 * 10^-4^ | 61.75 |
| 43 | Gross Income | Social | Numerical | 2.82 * 10^-4^ | 61.75 |
| 44 | Days since Most Recent GP HDL Measurement | Medical | Numerical | 2.71 * 10^-4^ | 49.49 |
| 45 | Highest GP Weight Measurement Filtered | Medical | Numerical | 2.47 * 10^-4^ | 29.77 |
| 46 | Days since Most Recent GP LDL Measurement | Medical | Numerical | 2.47 * 10^-4^ | 44.81 |
| 47 | Days since Highest GP Triglycerides Measurement | Medical | Numerical | 2.43 * 10^-4^ | 47.9 |
| 48 | Patient Owns Own Home | Social | Categorical | 2.30 * 10^-4^ | 61.75 |
| 49 | Highest GP Weight Measurement | Medical | Numerical | 2.14 * 10^-4^ | 29.91 |
| 50 | Days since Most Recent GP Triglycerides Measurement | Medical | Numerical | 2.08 * 10^-4^ | 48.03 |
| 51 | Number of Children in Household | Social | Numerical | 1.99 * 10^-4^ | 95.76 |
| 52 | Most Recent GP Weight Measurement Filtered | Medical | Numerical | 1.83 * 10^-4^ | 29.8 |
| 53 | Age of Oldest Child | Social | Numerical | 1.82 * 10^-4^ | 39.44 |
| 54 | Most Recent GP Cholesterol Measurement | Medical | Numerical | 1.74 * 10^-4^ | 50.77 |
| 55 | Days since Most Recent GP Cholesterol Measurement | Medical | Numerical | 1.73 * 10^-4^ | 50.98 |
| 56 | Income Compared to the European Poverty Line Category is Not One of Top 10 Most Frequent | Social | Categorical | 1.51 * 10^-4^ | 61.75 |
| 57 | Number of Persons in Household of “Other” Category (Non Partner, Non Child, Non Parent) | Social | Numerical | 1.50 * 10^-4^ | 95.76 |
| 58 | Most Recent GP Weight Measurement | Medical | Numerical | 1.44 * 10^-4^ | 29.85 |
| 59 | Most Recent GP HbA1c Measurement | Medical | Numerical | 1.41 * 10^-4^ | 3.14 |
| 60 | Days since Most Recent GP HbA1c Measurement | Medical | Numerical | 1.40 * 10^-4^ | 3.43 |
| 61 | One Person Household, Man Aged 67+ | Social | Categorical | 1.39 * 10^-4^ | 61.75 |
| 62 | Highest GP HbA1c Measurement Filtered | Medical | Numerical | 1.34 * 10^-4^ | 3.24 |
| 63 | Degree of Benefit Dependency of Private Households in the Reporting Year, Category is Not One of Top 10 Most Frequent | Social | Categorical | 1.10 * 10^-4^ | 61.75 |
| 64 | Number of Persons in Household is 2 | Social | Categorical | 9.59 * 10^-5^ | 61.75 |
| 65 | Percentile Group of Primary Income of Private Households is Not One of Top 10 Most Frequent | Social | Categorical | 9.37 * 10^-5^ | 61.75 |
| 66 | Highest Combined Hospital Weight Measurement | Medical | Numerical | 9.23 * 10^-5^ | 1.97 |
| 67 | GP LDL Measurement is Missing | Medical | Categorical | 8.34 * 10^-5^ | 100 |
| 68 | Inclusion Code is ICPC T82 | Other | Categorical | 7.46 * 10^-5^ | 100 |
| 69 | Number of Persons in Household | Social | Numerical | 6.61 * 10^-5^ | 95.76 |
| 70 | Days since Most Recent Hospital 1 LDL Measurement | Medical | Numerical | 6.54 * 10^-5^ | 0.23 |
| 71 | Household Composition Category is Not One of Top 10 Most Frequent | Social | Categorical | 6.51 * 10^-5^ | 61.75 |
| 72 | Highest Combined Hospital HDL Measurement | Medical | Numerical | 6.50 * 10^-5^ | 0.65 |
| 73 | 5th Percentile Group of Primary Income of Private Households | Social | Categorical | 6.45 * 10^-5^ | 61.75 |
| 74 | Most Recent Hospital 2 Weight Measurement | Medical | Numerical | 6.13 * 10^-5^ | 1.73 |
| 75 | 28th Percentile Group of Gross Income of Private Households | Social | Categorical | 6.13 * 10^-5^ | 61.75 |
| 76 | Patient’s Father’s Country of Birth is Suriname | Social | Categorical | 6.09 * 10^-5^ | 100 |
| 77 | 37th Percentile Group of Spendable Income of Private Households | Social | Categorical | 6.08 * 10^-5^ | 61.75 |
| 78 | Patient’s Father’s Country of Birth is The Netherlands | Social | Categorical | 5.98 * 10^-5^ | 100 |
| 79 | Spendable Income is between 99% and 100% of Policy Minimum in the Reporting Year | Social | Categorical | 5.90 * 10^-5^ | 61.75 |
| 80 | Patient’s Mother’s Country of Birth is the Dutch East Indies | Social | Categorical | 5.77 * 10^-5^ | 100 |
| 81 | Household is Not Part of the Target Population (Private Households with Observed Income) in at Least 2 of the 3 Preceding Years | Social | Categorical | 5.73 * 10^-5^ | 61.75 |
| 82 | Days since Highest GP HbA1c Measurement | Medical | Numerical | 5.57 * 10^-5^ | 3.24 |
| 83 | Main Benefit of the Household is Social Assistance Benefit | Social | Categorical | 5.51 * 10^-5^ | 61.75 |
| 84 | Highest Combined Hospital LDL Measurement | Medical | Numerical | 5.44 * 10^-5^ | 0.63 |
| 85 | Household Lives in Rental Property without Rent Allowance | Social | Categorical | 5.23 * 10^-5^ | 61.75 |
| 86 | Household Contains 2 Persons with Personal Income | Social | Categorical | 4.89 * 10^-5^ | 61.75 |
| 87 | Highest Hospital 2 Weight Measurement | Medical | Numerical | 4.75 * 10^-5^ | 1.76 |
| 88 | Highest Combined Hospital BMI Measurement | Medical | Numerical | 4.48 * 10^-5^ | 0.88 |
| 89 | Primary Household Income is Profit from Self-Employment | Social | Categorical | 4.34 * 10^-5^ | 61.75 |
| 90 | Inclusion Code is ICPC Y07 | Other | Categorical | 4.25 * 10^-5^ | 100 |
| 91 | 12th Percentile Group of Primary Income of Private Households | Social | Categorical | 4.17 * 10^-5^ | 61.75 |
| 92 | Most Recent Combined Hospital Height Measurement | Medical | Numerical | 4.07 * 10^-5^ | 1.07 |
| 93 | Further Differentiation of a Person Based on Dutch Background, First and Second Generation Migration Background Respectively | Social | Categorial | 4.00 * 10^-5^ | 99.3 |
| 94 | Highest Combined Hospital Glucose Measurement | Medical | Numerical | 3.86 * 10^-5^ | 1.97 |
| 95 | Degree of Benefit Dependency in Reporting Year is 0%, Household Receives No Benefits | Social | Categorical | 3.72 * 10^-5^ | 61.75 |
| 96 | Household Income is 65% to 66% of the European Poverty Line | Social | Categorical | 3.68 * 10^-5^ | 61.75 |
| 97 | Household Income is 73% to 74% of the European Poverty Line | Social | Categorical | 3.67 * 10^-5^ | 61.75 |
| 98 | Most Recent Combined Hospital HDL Measurement | Medical | Numerical | 3.40 * 10^-5^ | 0.65 |
| 99 | Income Compared to the Low-Income Threshold in the Reporting Year Category is Not One of Top 10 Most Frequent | Social | Categorical | 3.31 * 10^-5^ | 61.75 |
| 100 | 94th Percentile Group of Standardised Spendable Income of Private Households | Social | Categorical | 3.12 * 10^-5^ | 61.75 |
